# Targeting the Neuro-Immune Axis in Next-Generation Oncology: Discovery and Validation of β_2_-Adrenergic Blockade to Reverse Ecosystem-Wide Resistance

**DOI:** 10.32604/or.2026.083919

**Published:** 2026-08-13

**Authors:** Heng Xu, Jiaan Lu, Zizhang Wang, Jiayu Xu, Shihui Peng, Haiqing Chen, Qiang Cao, Qing Sun, Shangke Huang

**Affiliations:** 1 Clinical Medical College, Southwest Medical University, Luzhou, China; 2 School of Computer Science and Informatics, Cardiff University, Cardiff, UK; 3 Department of Public Health Laboratory Sciences, School of Public Health, Hengyang Medical School, University of South China, Hengyang, China; 4 Department of Orthopedics, The First Affiliated Hospital of Chongqing Medical University, Chongqing, China; 5 Faculty of Applied Sciences, Macao Polytechnic University, R. de Luís Gonzaga Gomes, Macao, China; 6 Department of Otolaryngology, QingPu Hospital Affiliated to Fudan University, Shanghai, China; 7 Department of Oncology, The Affiliated Hospital, Southwest Medical University, Luzhou, China

**Keywords:** Predictive biomarkers, multi-omics, sympathetic-immune axis, tumor ecosystem, immunotherapy resistance, β_2_-adrenergic receptor, clinical translation

## Abstract

Despite the potential of current cancer immunotherapies, tumor cells frequently evade immune surveillance by forming an immunosuppressive microenvironment, leading to treatment resistance. Current inquiry positions the sympathetic nervous system (SNS) at the forefront of tumor immunology as a critical driver of this immune evasion. This review delineates the cellular pharmacology of SNS-mediated immune regulation across the tumor ecosystem. Operating predominantly through the cyclic adenosine monophosphate-protein kinase A (cAMP-PKA) signaling axis, the SNS engages in bidirectional regulation with immune cells of the tumor microenvironment (TME). Norepinephrine and epinephrine interact with β_2_-adrenergic receptors (β_2_-ARs), triggering G-protein dissociation, adenylyl cyclase stimulation, and cAMP generation. This pharmacological cascade promotes the polarization of macrophages to the M2 phenotype, hinders dendritic cell maturation, impairs natural killer (NK) cell function, fosters the differentiation of regulatory T cells (Tregs), and suppresses the effector activity of CD8^+^ T cells. Consequently, we evaluate the translational prospects of repurposing β-blockers as potent anti-cancer immunomodulators. Preclinical evidence demonstrates that pharmacological blockade of β_2_-AR signaling can reverse these immunosuppressive effects, augmenting intratumoral CD8^+^ T-cell infiltration and enhancing the efficacy of immune checkpoint inhibitors (ICIs). Ultimately, engineering multimodal combination regimens—guided by multi-omics biomarker stratification—offers a novel trajectory to overcome resistance, restore therapeutic sensitivity, and advance precision cancer immunotherapy.

## 1 Introduction

The immune system suppresses tumorigenesis via a tripartite mechanism: clearance of oncogenic viruses, regulation of the inflammatory microenvironment [1], and cytotoxic killing mediated by recognition of tumor-associated antigens [2,3]. However, during the development and progression of tumors, tumor cells can evade immune surveillance by losing antigenicity [4], losing immunogenicity, and forming an immunosuppressive microenvironment [5]. In the tumor microenvironment (TME), the composition and functional state of infiltrating immune cells have a profound impact on tumor growth and patient prognosis. Increased infiltration of cytotoxic CD8^+^ T lymphocytes has been associated with improved clinical prognoses and prolonged overall survival in patients [6]. Conversely, a greater abundance of regulatory T cells (Tregs) correlates with poorer prognosis [7]. Moreover, M2-polarized macrophages exhibit immunosuppressive properties and actively promote tumor cell proliferation, invasion, and migration [8,9]. These research findings provide a significant theoretical foundation for the development and application of new tumor immunotherapies.

Conventional tumor immunology studies have predominantly concentrated on local cellular and molecular interactions, frequently neglecting the regulatory influence of the sympathetic nervous system (SNS). Recent evidence indicates that fibers of the SNS are capable of directly innervating tumor tissues [10,11] and establishing immune synapse-like structures modulated by immune cells [12,13]. These observations highlight a pivotal role of the SNS in shaping the immune landscape of the TME. These nerve fibers exert regulatory effects on immune cell recruitment and activity through the release of adrenergic neurotransmitters [14]. Notably, activation of the β_2_-adrenergic receptor (β_2_-AR) pathway represents a crucial mechanism that restricts T lymphocyte entry into tumor sites, thereby exerting a significant impact on tumor growth and advancement [10].

Therefore, the primary objective of this review is to systematically elucidate the pharmacological mechanisms by which the SNS drives tumor immune evasion via β_2_-AR signaling. Furthermore, we aim to construct a biomarker-driven, multimodal clinical translation framework to evaluate the repurposing of β-blockers as immunomodulatory adjuncts, with the ultimate goal of reversing ecosystem-wide resistance and enhancing cancer immunotherapy.

## 2 Foundations of SNS-Activated TME

Chronic stress, characterized by prolonged exposure to uncontrollable psychological or physiological stressors, triggers persistent compensatory responses in the neuroendocrine system, with the overactivation of the SNS being recognized as a central factor in driving stress-induced pathophysiological alterations. Chronic stress–driven activation of the SNS and its pro-tumor consequences are highly context dependent [15,16]. Distinct stress modalities—for example, psychological/emotional stress versus physical/physiological stress—may recruit partially different neural and endocrine pathways, thereby shaping SNS tone and the extent of sympathetic innervation within the tumor. Experimental models show that chronic restraint stress can increase intratumoral NE concentrations by several fold and is closely associated with the sprouting of sympathetic fibers into the tumor mass, indicating that stress-induced sympathetic remodeling is a core pathophysiological driver of tumor progression [17,18]. At the same time, these findings suggest that what is often labeled “chronic stress” should not be viewed as a simple binary state, but rather as a cumulative adrenergic load delivered to the TME, with both temporal (sustained versus transient catecholamine exposure) and spatial (local high-density nerve–immune contacts) dimensions. However, a quantitative framework that systematically integrates stress intensity, exposure duration, and the density of tumor-associated sympathetic innervation into a predictive model of immune suppression has not yet been established. Defining this “stress–innervation–immunosuppression” relationship is likely to be a foundational requirement for translating behavioral or autonomic interventions into precise, stage-appropriate anticancer strategies.

Chronic stress engages both the sympathoadrenal medullary (SAM) axis and the hypothalamic–pituitary–adrenal (HPA) axis, resulting in persistent release of NE and epinephrine (Epi) [17,19,20]. NE released by sympathetic nerve endings and Epi produced by the adrenal medulla act as key effectors through which the SNS modulates the TME [21]. These catecholamines exert local effects—either via systemic circulation or direct neural innervation of the tumor—interacting with cancer and immune cells in paracrine and autocrine fashions [22].

NE and Epi can interact with β_2_-AR expressed on tumor as well as immune cells, initiating downstream signaling pathways that engage mediators including vascular endothelial growth factor (VEGF) and matrix metalloproteinases (MMPs). This interaction promotes the shift of β_2_-AR from an inactive to an active conformation, facilitating downstream effects.

This shift triggers G-protein dissociation and activation of downstream effectors [23,24]. Activated β_2_-AR engages Gs proteins, causing GDP release from the Gαs subunit and its subsequent binding to GTP [25,26]. The released Gαs-GTP complex subsequently stimulates adenylyl cyclase (AC), promoting the conversion of ATP into cyclic AMP (cAMP) [27,28]. The generated cAMP binds to the regulatory domains of protein kinase A (PKA), inducing dissociation of its regulatory and catalytic subunits and thereby activating PKA [29].

Once activated, PKA phosphorylates multiple downstream substrates, thereby modulating cellular metabolism, gene expression, and physiological functions [27,30]. Given that cAMP signaling governs cell proliferation and differentiation, aberrant cAMP dynamics have been implicated in tumorigenesis [31]. Indeed, cAMP–PKA signaling regulates cancer cell growth, migration, invasion, and metabolism [27,32]. In preclinical models of hepatocellular carcinoma, lung cancer, breast carcinoma, glioma, and lymphoma, activation of the cAMP-mediated PKA pathway has been shown to enhance tumor cell proliferation, sustain survival, regulate motility, increase adhesiveness, and promote invasiveness [31,33]. Moreover, malignant traits such as invasion, migration, adhesion, and clonogenicity in medulloblastoma, ovarian carcinoma, colorectal carcinoma, breast carcinoma, and pituitary tumors are closely associated with cAMP/PKA signaling [30,34].

**Figure 1:**
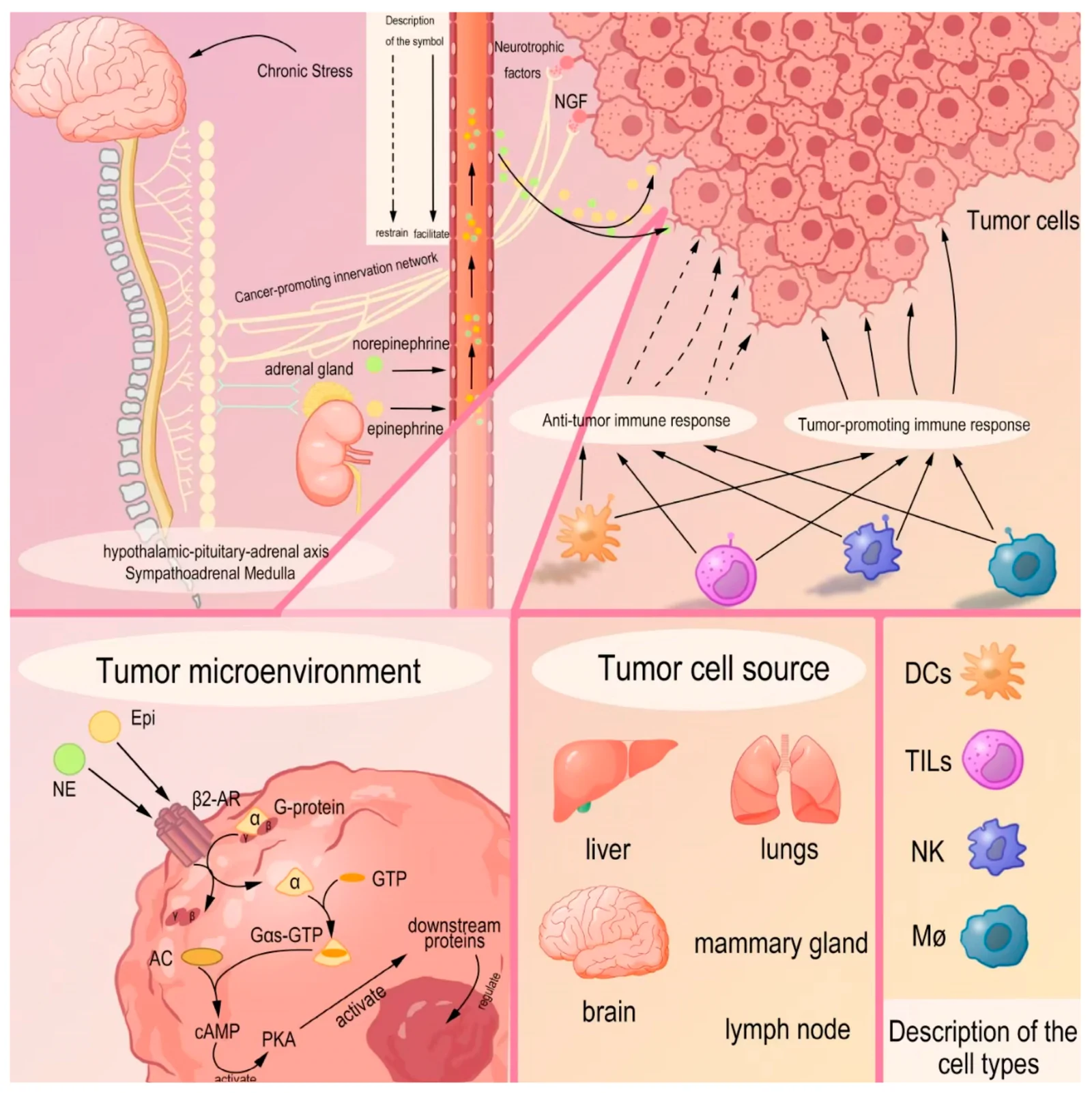
**Sympathetic nervous system (SNS)–β_2_-adrenergic signaling modulates tumor immunity.** Chronic stress activates the SNS and hypothalamic–pituitary–adrenal axis, promoting norepinephrine (NE) and epinephrine (Epi) release. These catecholamines act on β_2_-adrenergic receptors (β_2_-ARs) within the tumor microenvironment (TME), initiating the cAMP–PKA pathway. This suppresses dendritic cell maturation, impairs NK and CD8^+^ T-cell function, and promotes regulatory T cell (Treg) and M2 macrophage polarization. Abbreviations: DCs, dendritic cells; TILs, tumor-infiltrating lymphocytes; NK, natural killer; M∅, macrophages.

Beyond the direct effects on cancer cells, neural signaling also profoundly orchestrates the TME. Specifically, tumor-infiltrating lymphocytes (TILs), dendritic cells (DCs), natural killer (NK) cells, and macrophages express receptors for various neurotransmitters and neuropeptides, allowing them to sense neural cues and adjust immune responses in a context-dependent manner. Engagement of specific receptors can either induce pro-tumorigenic immunity or, when blocked, enhance antitumor immunity [35,36]. Concurrently, tumor cells within the TME may secrete neurotrophic factors—such as nerve growth factor (NGF) [18]—to increase sympathetic fiber infiltration, thereby establishing a pro-tumorigenic neural network [37] (Fig. 1).

## 3 Mechanisms of SNS-Mediated Immune Regulation.

While the primary focus of this review is the β_2_-AR signaling axis, it is critical to acknowledge that the SNS orchestrates tumor immunity through a broader repertoire of receptors, including α-adrenergic receptors (α-ARs) and dopamine receptors [16]. For instance, α-ARs expressed on myeloid cells can modulate cytokine production and macrophage polarization, while vascular α_1_-ARs influence tumor perfusion and subsequent immune cell infiltration. Similarly, dopamine—a precursor to NE and an independent neurotransmitter—can interact with dopamine receptors (DRs) on specific immune subsets to exert context-dependent immunomodulatory effects [38]. However, among these diverse pathways, the β_2_-AR is uniquely characterized by its near-ubiquitous expression across almost all immune subsets within the TME. Furthermore, chronic β_2_-AR activation is overwhelmingly recognized as the dominant neuro-immune driver of profound immune exhaustion and therapy resistance [18]. Therefore, to provide a mechanistically deep and clinically actionable framework, this review specifically concentrates on the β_2_-AR axis as the primary therapeutic vulnerability.

### 3.1 Effects on Innate Immune Cells

#### 3.1.1 Macrophages

Sympathetic neurotransmitters including NE and Epi engage β-adrenergic receptors on macrophages, enhancing the expression of immunosuppressive molecules—such as arginase-1 (ARG1), PD-L1, CD47, and the anti-inflammatory cytokine IL-10 [39,40,41]—while concurrently downregulating M1-related markers, evidenced by markedly reduced TNF-α levels [41,42]. This shifts macrophage polarization toward the M2 tumor-associated phenotype (TAMs), fostering an immunosuppressive microenvironment [43]. M2-polarized TAMs facilitate tumor progression via two principal mechanisms:

Inhibition of T-cell activation: Loss of co-stimulatory molecules (CD80/CD86/CD40) impairs T-cell priming [43], and high PD-L1 expression induces T-cell exhaustion [41]. Promotion of angiogenesis, invasion, and proliferation: M2 TAMs secrete VEGF and PDGF to drive neovascularization [44,45], release MMP-9 to degrade the extracellular matrix and facilitate metastasis [44], and transfer oncogenic miRNAs (miR-155-5p/miR-221-5p) via exosomes to accelerate tumor cell proliferation [18,44]. Collectively, these effects contribute to a protumorigenic immune landscape.

#### 3.1.2 Dendritic Cells (DCs)

##### Inhibition of Maturation and Antigen Presentation

Under homeostatic conditions, DC maturation depends on activation of the MAPK/NF-κB pathways, leading to up-regulation of MHC-II and CD80/CD86 and initiation of T-cell–mediated antitumor immunity [46,47]. SNS activation of β-AR, however, triggers the cAMP–PKA cascade, which suppresses MAPK/NF-κB signaling [48], thereby blocking DC maturation, diminishing antigen-presenting capacity, and resulting in suboptimal T-cell activation [49].

##### Cytokine Profile Reprogramming

Cytokine profile reprogramming entails alteration of cytokine expression patterns or signaling pathways to influence cell differentiation, function, and fate. Mature DCs typically secrete IL-12 to promote Th1 differentiation and cell-mediated immunity [50,51]. SNS signaling down-regulates DC IL-12 production—weakening Th1 responses—while increasing release of immunosuppressive cytokines such as IL-10, which drives regulatory T-cell (Treg) differentiation and further suppresses T-cell activation [52]. Future research should determine whether these cytokine alterations are reversible via selective β-AR inhibition or if combined strategies (e.g., DC-targeted vaccines plus adrenergic blockade) are necessary to restore robust proinflammatory signaling in tumor-infiltrating DCs.

#### 3.1.3 NK Cells

##### Direct Inhibition

SNS-derived catecholamines inhibit NK cell activity [13], reducing secretion of Th1-type cytokines (IL-12, IFN-γ), impairing cytotoxic function, and elevating Th2-type IL-10 production [53], thereby facilitating tumor immune escape and proliferation.

##### TME-Mediated Synergistic Suppression

Tumor cells secrete immunomodulatory factors such as TGF-β and PGE_2_ [54]. TGF-β down-regulates activating NK receptors (e.g., NKG2D, NKp30) while up-regulating inhibitory receptors (e.g., NKG2A, KIR) [55,56,57], further impairing NK cell function and undermining tumor immune surveillance.

### 3.2 Effects on Adaptive Immune Cells

#### 3.2.1 CD8^+^ T Cells

##### Direct Inhibition of Activation and Metabolism

Activation of β_2_-adrenergic receptors (β_2_-AR) on CD8^+^ T cells engages the cAMP/PKA axis, which inhibits ZAP70 phosphorylation and CD3ζ-chain signal transduction, while upregulating the checkpoint receptor PD-1; this collectively suppresses CD28-mediated co-stimulatory signaling and T-cell activation [58].

##### Suppression of Key Cytokine Secretion

β_2_-AR signaling significantly reduces the secretion of IL-2 and other tumor cytokines, thereby inhibiting T cell proliferation and reducing their cytotoxic function [58,59,60]. The reduction in IFN-γ interferes with APC production of IL-12 and weakens the Th1 response. Additionally, reduced MHC-I/II expression on target cells decreases the efficiency of antigen presentation [61], and reduced MHC-I/II expression also disrupts the balance between pro-tumor and antitumor immunity in the TME [62]. Recent studies have revealed that SNS signaling, via the cAMP-PKA axis, exerts more than just an acute suppression of TCR signaling and other immediate effects. It also shapes the core molecular profile of T cell exhaustion through epigenetic and transcriptional reprogramming, thus driving irreversible functional exhaustion in CD8^+^ T cells [58,63]. As established by foundational original reports, the epigenetic landscape of exhausted T cells is strictly governed by precise regulatory events, primarily driven by the overexpression of the transcription factor TOX, alongside the profound inhibition of TCF-1, a crucial factor responsible for maintaining stem-like properties and self-renewal [64,65,66,67]. In the context of adrenergic stress, the sympathetic signaling cascade intersects with these established epigenetic programs. Specifically, activated PKA phosphorylates the transcription factor CREB, which can subsequently bind to relevant regulatory regions to further promote the expression of exhaustion-associated profiles, including TOX. Meanwhile, the cAMP-PKA axis antagonizes key survival and self-renewal pathways (such as Wnt/β-catenin), leading to a sustained repression of TCF-1 expression. This “bidirectional regulation”—characterized by TOX upregulation and TCF-1 downregulation—fundamentally drives CD8^+^ T cells toward a state of functional decline, stripping them of their proliferative potential and cytotoxic activity.

These findings provide a deeper mechanistic explanation for the action of β_2_-AR blockers, showing that their intervention goes beyond reversing transient signal suppression. They may also block pathogenic transcriptional programs, fundamentally reshaping T cell fate and restoring their effector functions and metabolic adaptability. This theoretical foundation paves the way for the combination of β_2_-AR blockade with immune checkpoint inhibitors (ICIs) to fundamentally rebuild T cell function.

#### 3.2.2 Regulatory T Cells (Tregs)

β_2_-AR activation modulates transcriptional control via the cAMP/PKA pathway: PKA phosphorylates CREB, promoting its nuclear translocation and binding to the Foxp3 promoter, thus enhancing Foxp3 transcription and driving Treg differentiation and maturation [68,69]. Tregs secrete anti-inflammatory cytokines—including IL-10, IL-35, and TGF-β—to inhibit DC function and foster conversion of naïve T cells into additional Tregs [70,71]. Elevated Treg CTLA-4 binds CD80/CD86 on DCs, blocking CD28 co-stimulation and reducing effector T-cell activation and proliferation [72]. In the TME, high Treg-to-effector T-cell ratios correlate with poorer clinical outcomes across multiple malignancies [71,73].

#### 3.2.3 Cytotoxic T Lymphocytes (CTLs)

##### Cytokine-Mediated Negative Feedback and Metabolic Inhibition

When memory CD8^+^ T cells are re-exposed to antigen, they secrete IL-2 and IFN-γ. IL-2 promotes upregulation of β_2_-adrenergic receptors (β_2_-AR) on effector T cells; nevertheless, stimulation of β_2_-AR signaling subsequently suppresses IL-2 production, establishing an autoinhibitory negative feedback mechanism [74]. In addition, β_2_-AR activation impairs mitochondrial function in CD8^+^ T cells, disrupting the metabolic reprogramming essential for full activation [63].

##### Impaired Trafficking and Microenvironmental Synergy

SNS-induced upregulation of trophic factors and chemokines (e.g., CXCL12) enhances adhesion between tumor cells and nerve fibers, thereby hindering CTL homing to neoplastic sites [18] (CAF activation is also influenced by chemokines such as CXCL12, which further modulates the TME and immune cell infiltration) [75]. Within the TME, elevated PD-1 on CTLs engages PD-L1, activating SHP1/SHP2 phosphatases and dampening TCR signaling [76]. Moreover, interactions between the pro-survival protein Bcl-2 and the apoptotic mediator Bim in PD-L1^+^ cells promote CD8^+^ T-cell apoptosis, further compromising CTL effector function and reducing tumor-cell lysis [76,77] (Table 1).

**Table 1:** Detailed SNS–immune interaction table.

Immune Cell Type	SNS/β_2_-AR Activation Effects	Key Signaling Pathways	Functional Consequence	Relevant Markers/Molecules
Macrophages (MØ)	Promotes M2 polarization; upregulates ARG1, IL-10, CD47, PD-L1; suppresses TNF-α and co-stimulatory molecules (CD80/CD86/CD40)	β_2_-AR → Gs → AC → cAMP → PKA	Supports tumor growth, metastasis, ECMremodeling, and immune escape	ARG1, IL-10, PD-L1, CD47, CD80, CD86, MMP-9, VEGF
Dendritic Cells (DCs)	Blocks maturation via cAMP–PKA; downregulates MAPK/NF-κB pathway; reduces MHC-II and CD80/CD86 expression	β_2_-AR → cAMP → PKA→↓MAPK/NF-κB	Supports tumor growth, metastasis, ECMremodeling, and immune escape	MHC-II, CD80/CD86, IL-12, IL-10, MAPK, NF-κB
Natural Killer Cells (NK)	Suppresses IFN-γ and IL-12 secretion; increases IL-10; downregulates activating receptors (NKG2D, NKp30) and upregulates inhibitory ones (NKG2A, KIR)	β_2_-AR → cAMP → PKA; also influenced by TGF-β, PGE_2_ in TME	Impaired cytotoxicity and immune surveillance, enhanced tumor tolerance	IFN-γ, IL-12, IL-10, NKG2D, NKp30, KIR, NKG2A
CD8^+^ T Cells	Inhibits ZAP70 and CD3ζ phosphorylation; impairs CD28 signaling; reduces IL-2 and IFN-γ; increases PD-1 expression	β_2_-AR → cAMP → PKA→↓TCR signaling, ↑PD-1	Reduces effector function, proliferation, and antigen recognition	ZAP70, CD3ζ, CD28, IL-2, IFN-γ, PD-1, MHC-I/II
Regulatory T Cells (Tregs)	Activates CREB via PKA; enhances Foxp3 transcription; increases IL-10, IL-35，TGF-β; promotes CTLA-4 expression and DC suppression	β_2_-AR → cAMP → PKA → CREB → Foxp3	Creates highly suppressive immune environment, linked to poor prognosis	CREB, Foxp3, IL-10, IL-35, TGF-β, CTLA-4
Cytotoxic T Lymphocytes (CTLs)	Forms a self-inhibitory loop: IL-2 upregulates β_2_-AR, which suppresses further IL-2 secretion. Disrupts mitochondrial metabolism; increases PD-1 engagement and apoptosis via Bcl-2/Bim interaction	β_2_-AR → cAMP → PKA; PD-1/PD-L1 → SHP1/SHP2	Dysfunction of CTL cells; reduced tumor cell clearance	IL-2, IFN-γ, β_2_-AR, CXCL12, PD-1, PD-L1, SHP1, SHP2, Bcl-2, Bim

## 4 Sympathetic Nerve Fibers and Tumors

The regulation of tumor immunity by β_2_-AR signaling is highly dynamic, strictly dictated by temporal duration, local hormone concentrations, receptor expression profiles, and spatial heterogeneity within the TME. Temporally, the duration of action fundamentally determines the immunological outcome: acute physiological stress induces transient catecholamine spikes that can mobilize immune cells, whereas chronic stress maintains persistently elevated NE and Epi concentrations. This sustained adrenergic load shifts the balance toward long-term immune exhaustion and tumor tolerance [18]. Spatially, sympathetic innervation and the resultant neurotransmitter gradients are highly uneven. Nerve fiber density is frequently enriched at the tumor’s invasive margins and perivascular niches, whereas the hypoxic tumor core remains relatively denervated, creating localized “adrenergic hotspots” [78]. Furthermore, the immunomodulatory impact is heavily dependent on receptor expression and local hormone concentration. Target cells within the TME exhibit heterogeneous β_2_-AR expression; for example, M2-like TAMs, Tregs, and exhausted CD8^+^ T cells positioned near these nerve terminals robustly upregulate β_2_-ARs, rendering them exquisitely sensitive to even subtle fluctuations in local catecholamine concentrations [79]. Consequently, this spatiotemporal heterogeneity dictates that SNS-immune interactions form a progressively evolving ecosystem, resulting in marked differences among different patients and tumor types [80,81].

Reflecting this complex ecosystem, the presence and functional impact of SNS fibers within tumor tissues display marked tumor-type specificity [11]. Broadly, intratumoral nerves may be classified as pro-tumorigenic, antitumorigenic, or dual-function fibers. In many solid tumors, sympathetic fibers are recruited and driven by tumor-derived neurotrophic factors—such as NGF and brain-derived neurotrophic factor (BDNF)—and infiltrate the TME via perivascular routes or through adjacent stromal tissue [18].

In prostate cancer, for example, chronic sympathetic adrenergic activity has been linked to tumor initiation and early growth, whereas cholinergic (parasympathetic) input has been associated with the promotion of local invasion and distant dissemination in later stages [82,83,84]. This indicates that tumor progression is not governed by sympathetic signaling alone, but rather emerges from a coordinated but imbalanced autonomic network in which both sympathetic and parasympathetic branches remodel the TME to favor immune suppression, tissue remodeling, and metastatic competency [85].

In gastric and colorectal malignancies, the role of the SNS remains contentious, with evidence supporting both tumor-promoting and tumor-suppressive functions [86,87].

## 5 Therapeutic Strategies and Applications

### 5.1 Prospects for β-Adrenergic Receptor Blockade

The role of sympathetic adrenergic signaling in cancer progression is strikingly tissue specific. In prostate cancer, sympathetic input has been implicated as a driver of early tumor growth and local progression [82], whereas in certain gastric and colorectal settings its net effect remains controversial and may even include context-dependent inhibitory influences [86]. Pancreatic ductal adenocarcinoma, by contrast, exhibits a highly desmoplastic stroma that physically limits neural infiltration, giving rise to a relatively “low-innervation” phenotype in which sympathetic fibers have restricted access to the malignant compartment [85].

Despite this spatial and anatomical heterogeneity, β_2_-AR signaling appears to act as a conserved immunosuppressive node across tumor types: it attenuates CD8^+^ T cell–mediated cytotoxicity, constrains effector T-cell infiltration and promotes dysfunctional/exhausted phenotypes in tumor-infiltrating lymphocytes [58]. This conserved β_2_-AR–dependent brake on antitumor immunity provides the biological rationale for exploring β-blockers as immunological co-therapeutics, including reports of synergy with immune checkpoint inhibitors (ICIs) in melanoma models [88]. However, any clinical deployment of β-blockade is unlikely to be uniformly effective across all malignancies. Its benefit will almost certainly depend on multiple modifiers, such as intratumoral sympathetic nerve density and remodeling, the adrenergic receptor expression profile on tumor and immune cells, and the availability of compensatory non–β_2_-AR pathways. These considerations argue for biomarker-guided patient stratification, rather than assuming that β-blockers are broadly applicable in a tumor-agnostic manner.

Successful integration of β-blockade into cancer immunotherapy further hinges on two unresolved clinical variables: dose and timing.

*Dose.* Most dosing information derives from cardiovascular indications, but those regimens may not correspond to the optimal immunomodulatory window. Preclinical data suggest that comparatively low doses of β-blockers can restore T-cell metabolic fitness, cytotoxic function and intratumoral infiltration [89], potentially relieving sympathetic suppression of antitumor immunity while avoiding overt haemodynamic toxicity. Early-phase clinical studies will therefore need to prioritize immunological readouts—such as CD8^+^ T-cell reinvigoration, increased effector infiltration and decreased suppressive myeloid/Treg populations—rather than relying solely on traditional cardiovascular tolerability windows.

*Timing.* The therapeutic window may also be indication specific. Neoadjuvant administration could, in principle, remodel an immunosuppressive TME before surgery. Adjuvant use may help eradicate minimal residual disease and reduce recurrence risk, consistent with epidemiological trends suggesting improved outcomes in certain breast cancer cohorts [90]. In advanced disease, concurrent β-blockade plus ICIs [91] aims to overcome immune exhaustion and checkpoint resistance in established lesions. Determining which of these windows is optimal for which tumor contexts is a central objective for prospective, stratified clinical trials.

Finally, safety cannot be treated as an afterthought. Combining β-blockers with ICIs raises a plausible risk of amplifying immune-related adverse events (irAEs), including life-threatening toxicities such as myocarditis and pneumonitis. Emerging evidence indicates that patients receiving ICIs alongside β-blockade warrant intensified, prospective safety monitoring [91]. The theoretical basis is straightforward: β-adrenergic signaling exerts a tonic suppressive influence on effector T cells, whereas ICIs remove intrinsic checkpoint restraints. Dual removal of these restraints could heighten systemic immune activation [92], improving tumor control but also increasing autoimmunity risk. We therefore argue that immune-toxicity surveillance and management protocols must be embedded as core design elements in β-blocker/ICI combination trials, rather than handled reactively.

### 5.2 Multimodal Combination Therapy Strategies

Short-term SNS hyperactivity can maintain cardiovascular homeostasis, whereas chronic activation exerts detrimental effects on cardiac and circulatory systems [93,94]. As a baseline intervention, behavioral modifications such as aerobic exercise and meditation have demonstrated efficacy in dampening resting SNS overactivity and lowering plasma NE levels [95,96,97,98,99]. Concurrently, conventional multimodal approaches—such as combining immunotherapy with radiotherapy or chemotherapy—have yielded synergistic effects in various solid tumors [100,101,102,103,104,105,106].

Although behavioral modifications and conventional treatments can effectively lower systemic adrenergic stress, their broad effects often fail to overcome the dense, localized neural networks protecting aggressive tumors. Translating the emerging field of “cancer sympatheology” into actual patient benefits requires us to move past broad theoretical concepts. Instead, future combination regimens must specifically target and disrupt the neuro-immune signaling loop within the tumor bed. To address this clinical gap, we outline three concrete, mechanism-driven strategies to guide future trial designs:

*Spatiotemporal sequencing of pharmacological blockade.* Simply administering β-blockers simultaneously with immune checkpoint inhibitors (ICIs) may yield suboptimal results due to pre-existing fibrotic barriers. We propose a “priming-then-activation” sequential strategy. In highly desmoplastic tumors, a 7-to-14-day “run-in” phase of non-selective β-blockade (e.g., propranolol) could inhibit adrenergic-driven stromal remodeling and alleviate local hypoxia prior to ICI administration [107,108]. Once this “neural-niche” is physically and metabolically dismantled, the subsequent introduction of anti-PD-1/PD-L1 therapy can achieve maximum intratumoral penetrance and CD8^+^ T cell reinvigoration.

*Biomarker-driven multi-omics stratification.* Not all patients require neuro-immune blockade. Implementing predictive modeling through multi-omics data mining is essential to guide multimodal therapy. By integrating bulk and single-cell RNA sequencing data, researchers can construct a specific “Neuro-Immune Risk Score” based on baseline sympathetic nerve density and β_2_-AR expression profiles on intratumoral macrophages and exhausted T cells [109]. Funneling only high-risk patients into intensive β-blockade + ICI combination trials will optimize response rates and prevent unnecessary systemic irAEs in low-risk cohorts.

**Figure 2:**
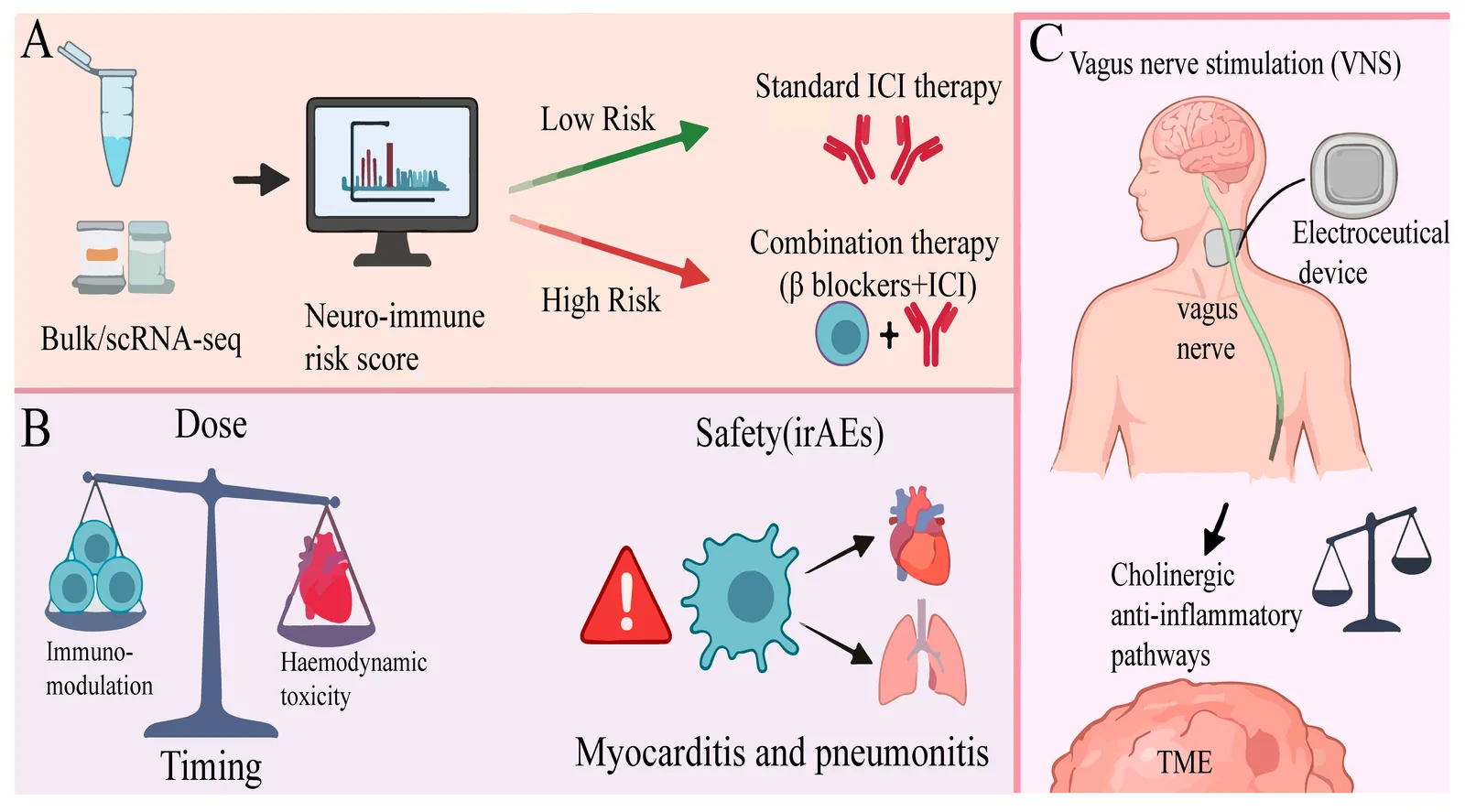
**Clinical translation framework and multimodal combination strategies targeting the neuro-immune axis.** (**A**) Biomarker-driven multi-omics stratification: By integrating bulk and single-cell RNA sequencing data, researchers can construct a specific “Neuro-Immune Risk Score” to funnel high-risk patients into intensive combination trials while preventing unnecessary systemic irAEs in low-risk cohorts. (**B**) Optimization of dose, timing, and safety: Successful integration of β-blockade hinges on balancing these critical variables. Optimized low dosing can restore T-cell metabolic fitness and cytotoxic function while avoiding overt hemodynamic toxicity. Concurrently, immune-toxicity surveillance must be embedded as core design elements to monitor the plausible risk of amplified immune-related adverse events (irAEs). (**C**) Targeted electroceuticals and TME-responsive nanomedicine: Evaluating non-invasive vagus nerve stimulation (VNS) represents a promising adjunctive strategy to synergistically counterbalance sympathetic overdrive. Furthermore, this can be integrated with intelligent lipid nanoparticles to release payloads selectively within hyper-innervated tumor margins. Abbreviations: TME, tumor microenvironment; ICI, immune checkpoint inhibitor.

*TME-responsive nanomedicine and targeted electroceuticals.* Systemic administration of β-blockers frequently causes dose-limiting cardiovascular toxicities. Drawing on the success of intelligent delivery vehicles, we propose the engineering of TME-responsive nanomedical platforms. Specifically, MMP-cleavable or hypoxia-responsive lipid nanoparticles co-loaded with β_2_-AR antagonists and ICIs could remain inert systemically but release payloads selectively within the hyper-innervated tumor margins [110,111]. Furthermore, these localized pharmacological approaches can be integrated with advanced neuromodulation techniques. Evaluating non-invasive vagus nerve stimulation (VNS) to activate cholinergic anti-inflammatory pathways represents a promising adjunctive strategy to synergistically counterbalance sympathetic overdrive and reprogram the immunosuppressive TME [112] (Fig. 2).

## 6 Conclusion

The SNS forms structured neuro–immune synapses with immune cells via tumor-innervating fibers, releasing NE and Epi that act on β_2_-AR to exert multifaceted regulatory effects on antitumor immunity. This review has synthesized evidence demonstrating that β_2_-AR activation engages the Gs protein–cAMP–PKA axis to modulate immune cell function. Tumor-derived neurotrophic factors can recruit sympathetic fibers to establish localized neural networks [18,37], and SNS-mediated regulation of the TME exhibits tissue specificity. In summary, these findings demonstrate the potential of targeting sympathetic nerve pathways as an adjunctive measure in cancer immunotherapy.

## 7 Future Directions

However, along with the advancement of β-AR-targeted therapies, the powerful adaptability of the tumor ecosystem must be acknowledged. Long-term inhibition of β_2_-AR signaling could not only undermine its direct tumor-promoting effects but also trigger compensatory neural signaling reprogramming, leading to the activation of other adrenergic, dopaminergic, or cholinergic pathways. For instance, in prostate cancer models, β_2_-AR blockade has been shown to upregulate α_1_-adrenergic receptor activity, maintaining tumor survival via the IP3/DAG-PKC pathway, while also shifting β_2_-AR signaling from the Gs-cAMP-PKA axis to the Gi-Src axis, which enhances the invasive phenotype. Simultaneously, cholinergic signaling from the paraSNS, alongside potential dopamine-mediated immunomodulation, may be abnormally activated, creating immune-suppressive synergies across neuro-branches and forming new immune escape routes at multiple levels. This compensatory potential suggests that adrenergic signaling may function as a ‘‘double-edged sword’’ in immune regulation.

Given these complexities, future therapeutic strategies should consider synergistic interventions targeting multiple neurotransmitter pathways. Additionally, dynamic biopsy samples from patients before and after treatment should be utilized to perform comprehensive neural molecular mapping, enabling real-time monitoring of adaptive resistance mechanisms. Nevertheless, significant challenges remain. While the potential of β-adrenergic receptor blockade in cancer immunotherapy is clear, the dynamic regulation of β_2_-AR signaling across discrete immune-cell subsets requires further elucidation. Techniques such as single-cell RNA sequencing and spatial transcriptomics will be crucial to understanding the dual roles of SNS signaling in various cancers, such as gastric and colorectal cancers, as well as the pathways through which SNS fibers influence the bone marrow microenvironment in hematologic malignancies.

To address these challenges, future studies should leverage organoid models and engineered neural–immune co-cultures to reconstruct neurotransmitter gradients and map SNS-driven regulatory networks at single-cell resolution. Advancing this field will require interdisciplinary collaboration—not only to identify reliable biomarkers of SNS activity but also to design clinical trials integrating β-adrenergic receptor blockade with immune checkpoint inhibitors, and to develop targeted delivery systems tailored to the TME. Furthermore, the successful clinical translation of these neuro-targeted immunotherapies hinges on overcoming significant pharmacological and safety hurdles. Establishing rigorous immune-toxicity surveillance protocols and identifying reliable, non-invasive circulating biomarkers will be indispensable for monitoring real-time therapeutic responses and mitigating adverse events. Moreover, exploring vagal nerve stimulation in combination with chemoradiotherapy represents an exciting avenue for neuro-immunomodulation. Iterative preclinical and clinical innovations will ultimately unify sympathoneural biology, immunology, and oncology into a cohesive framework, establishing the emerging field of ‘‘cancer sympatheology’’ and providing an expandable technological substrate for personalized immune interventions that intercept tumor initiation, progression, and dissemination.

## Data Availability

Not applicable.

## Abbreviations

Term	Interpretation
TNBC	Triple-Negative Breast Cancer
AC	Adenylyl Cyclase
AD	Alzheimer’s Disease
AKT	Protein Kinase B
ARG1	Arginase-1
ATP	Adenosine Triphosphate
BDNF	Brain-Derived Neurotrophic Factor
Bcl-2	B-cell Lymphoma 2
cAMP	Cyclic Adenosine Monophosphate
CAR-T	Chimeric Antigen Receptor T cell
CD28	Cluster of Differentiation 28
CD8	Cluster of Differentiation 8
CD80	Cluster of Differentiation 80
CD86	Cluster of Differentiation 86
CTLA-4	Cytotoxic T-Lymphocyte–Associated Protein 4
CREB	cAMP Response Element-Binding Protein
CXCL12	C-X-C Motif Chemokine Ligand 12
DCs	Dendritic Cells
DHEA	Dehydroepiandrosterone
ER	Estrogen Receptor
GABA	Gamma-Aminobutyric Acid
GCP	Good Clinical Practice
GS	Glutamine Synthetase
IFN-γ	Interferon-γ
IKK	IκB Kinase
IL-2	Interleukin-2
IL-10	Interleukin-10
IL-12	Interleukin-12
IL-35	Interleukin-35
LVEF	Left Ventricular Ejection Fraction
M2	Macrophage Type 2
MAPK	Mitogen-Activated Protein Kinase
MHC-I	Major Histocompatibility Complex Class I
MHC-II	Major Histocompatibility Complex Class II
NK	Natural Killer
NE	Norepinephrine
NF-κB	Nuclear Factor kappa-light-chain-enhancer of activated B cells
NKG2D	Natural Killer Group 2D
PD-1	Programmed Cell Death Protein 1
PD-L1	Programmed Death-Ligand 1
PE	Pulmonary Embolism
PGE_2_	Prostaglandin E2
PKA	Protein Kinase A
RBC	Red Blood Cells
SHP1	Src Homology Region 2 Domain-Containing Phosphatase-1
SNS	Sympathetic Nervous System
TCR	T-cell Receptor
TGF-β	Transforming Growth Factor-beta
TILs	Tumor-Infiltrating Lymphocytes
TME	Tumor Microenvironment
Treg	Regulatory T Cells
TSA	Tumor-Specific Antigen
VEGF	Vascular Endothelial Growth Factor
ZAP70	ζ-chain–Associated Protein Kinase 70
